# MEK1/2 inhibitor inhibits neointima formation by activating miR-126-3p/ C-X-C motif chemokine ligand 12 (CXCL12)/C-X-C motif chemokine receptor 4 (CXCR4) axis

**DOI:** 10.1080/21655979.2022.2063496

**Published:** 2022-04-29

**Authors:** Yali Yan, Mengmeng Zhu, Jialing Ma, Xiaoyu He, Xiaoxiao Yang, Hongmei Xu, Meixiu Jiang, Shuang Zhang, Yajun Duan, Jihong Han, Yuanli Chen

**Affiliations:** a Key Laboratory of Metabolism and Regulation for Major Diseases of Anhui Higher Education Institutes, School of Food and Biological Engineering, Hefei University of Technology, Hefei, Anhui, China; b The Institute of Translational Medicine, the National Engineering Research Center for Bioengineering Drugs and the Technologies, Nanchang University, Nanchang, Jiangxi, China; c College of Life Sciences, Key Laboratory of Medicinal Chemical Biology, Key Laboratory of Bioactive Materials of Ministry of Education, Nankai University, Tianjin, Hebei, China

**Keywords:** Neointima formation, ERK1/2, miR-126-3p, RGS16, endothelium repair

## Abstract

Endothelial dysfunction is an initial and essential step in vascular-remodeling diseases, including atherosclerosis and neointima formation. During vascular remodeling, activated endothelial cells can release pro-inflammatory factors that promote phenotypic switching of vascular smooth muscle cells (VSMCs) to the proliferative phenotype. We previously reported that MEK1/2 inhibitor, U0126, has a protective effect on the development of atherosclerosis and vascular calcification. However, the effect of MEK1/2 inhibitors on neointimal formation and the underlying mechanism is not fully understood. We determined that MEK1/2 inhibitor reduced carotid artery ligation-induced neointimal formation, while increased collagen and elastin levels and vascular integrality. Mechanistically, MEK1/2 inhibitor or ERK1/2 siRNA increased miR-126-3p level in endothelial cells, thereby inhibiting expression of regular of G-protein signaling 16 (RGS16), a miR-126-3p target gene, to activate the C-X-C motif chemokine ligand 12 (CXCL12)/C-X-C motif chemokine receptor 4 (CXCR4) signaling pathway. Accordingly, miR-126-3p was also increased by U0126 in serum and carotid artery. RGS16 was inhibited while CXCR4 and CXCL12 was increased by U0126 in neointimal areas, especially in the endothelium. Moreover, similar results were observed in atherosclerotic plaques of high-fat diet-fed apolipoprotein E deficiency (apoE^−/−^) mice. In addition, vascular cell adhesion molecule 1 (VCAM-1), another miR-126-3p target gene, was reduced by U0126 in the neointimal areas, resulting reduced monocytes/macrophages accumulation. Taken together, our results indicate that MEK1/2 inhibitor can reduce neointima formation by activating endothelial miR-126-3p production to facilitate endothelium repair while reduce monocyte adhesion/infiltration.

## GRAPHICAL ABSTRACT

**Figure:**
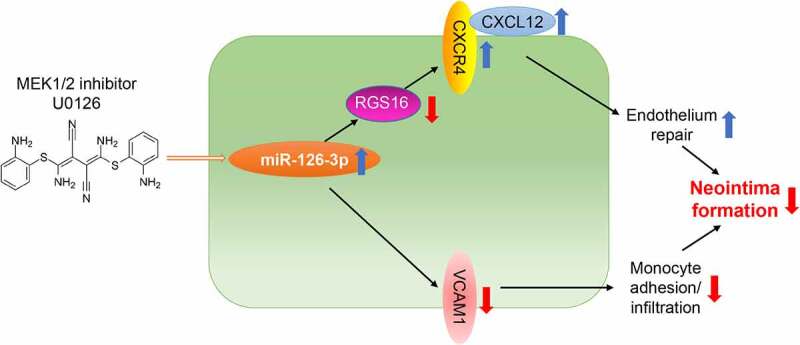

## Highlights

- MEK1/2 inhibitor reduces carotid artery ligation-induced neointima formation;
- MEK1/2 inhibitor induces miR-126-3p expression in endothelial cells;
- MEK1/2 inhibitor reduces RGS16 expression to activate CXCL12/CXCR4 signaling.
- MEK1/2 inhibitor reduces VCAM1 expression to reduce monocyte adhesion/infiltration.

## Introduction

Vascular remodeling is associated with dysfunctions of endothelial cells (ECs) and differentiation/proliferation of VSMCs, which contribute to the formation of in-stent restenosis, atherosclerosis, and vein graft failure [1]. In the initial step of vascular remodeling, ECs activation and dysfunction promote monocytes adhesion, which release various cytokines and inflammatory mediators to induce the phenotypic transformation and migration of VSMC to the intima. The transformed VSMCs in the intima further proliferate and synthesize a large number of extracellular matrix, which leads to the formation of neointima [2].

MicroRNAs (miRNAs), a type of endogenous non-coding small RNA with a length of 19–24 nt, mainly regulate target gene expression by binding to the 3′ untranslated regions (3′UTRs) of mRNAs, thereby causing translational repression and/or mRNA destabilization [3]. Studies have found that miRNAs play an important role in cardiovascular disease, such as heart failure, angiogenesis, hypertension, and atherosclerosis. MiR-126-3p is a miRNA with a length of 22 nucleotides, and its coding gene is located in the intron of epidermal growth factor-like domain multiple 7 (EGFL7). EGFL7 is a secreted angiogenic factor, which was mainly expressed in endothelial cells to regulate angiogenesis. Studies also showed that miR-126-3p can regulate angiogenesis by affecting the expression of vascular endothelial growth factor (VEGF) [4–6]. Moreover, miR-126-3p activates endothelial C-X-C motif chemokine ligand 12 (CXCL12)/C-X-C motif chemokine receptor 4 (CXCR4) signaling pathway by targeting regular of G-protein signaling 16 (RGS16), which can play a role in endothelial repair [6]. Additionally, miR-126-3p also reduced palmitate-induced ECs apoptosis and monocyte adhesion to ECs by targeting tumor necrosis factor receptor-associated factor 7 and vascular cell adhesion molecule 1 (VCAM-1), respectively [7,8].

Extracellular signal-regulated kinases 1/2 (ERK1/2) are a subfamily of mitogen-activated protein kinase (MAPK) signaling pathway that regulate cell growth and differentiation. ERK1/2 have been proven to involve in multiple diseases including cancers, bone formation and resorption, and cardiovascular disease. Inhibition of ERK1/2 by MEK1/2 inhibitor reduced atherosclerosis, which was associated with SMC contractive gene expression [9]. Furthermore, we previously reported that inhibition of ERK1/2 can inhibit vascular calcification by increasing endothelial miR-126-3p maturation and secretion, thereby inhibiting SMC osteoblast-like cell differentiation [10]. Moreover, ERK1/2 has been shown to promote the proliferation of VSMCs that is associated with the development of restenosis. ERK1/2 activation was detected in SMCs of rat carotid artery balloon injury and human atherosclerotic lesions [10,11]. However, whether MEK1/2 inhibitors affect neointimal formation by regulating miR-126-3p is unclear.

In this study, we proposed that MEK1/2 inhibitor can inhibit neointima formation by activating miR-126-3p expression to facilitate endothelium repair. We aimed to investigate the potential role of MEK1/2 inhibitor, U0126, in regulating miR-126-3p/RGS16/CXCL12/CXCR4 axis. We firstly examined the detailed actions of U0126 on vascular remodeling. Mechanistically, we determined that MEK1/2 inhibitor attenuated the expression of endothelial RGS16 to activate CXCL12/CXCR4 pathway by enhancing miR-126-3p expression. Our study provided a theoretical basis for drug therapy of in-stent restenosis.

## Methods

### Reagents

Rabbit anti-SMA and rat anti-MOMA2 polyclonal antibodies were purchased from Santa Cruz Biotechnology (Dallas, Texas, USA). Rabbit anti-CXCR4, GAPDH, RGS16, VCAM-1, and SM22α polyclonal antibodies and mouse anti-CD31 monoclonal antibody were purchased from Proteintech Group (Chicago, IL, USA). Rabbit anti-CXCL12 polyclonal antibody was purchased from Novus Biologicals (USA). U0126 (a MEK1/2 inhibitor) was purchased from LC Laboratories (Woburn, MA, USA). miR-126-3p antagomir and ERK1/2 siRNA were purchased from Guangzhou Ribobio (Guangzhou, Guangdong, China). Triglyceride (TG) kit, Total cholesterol (TC) kit, High-density lipoprotein Cholesterol (HDL-C) kit and Low-density lipoprotein cholesterol (LDL-C) kit were purchased from Medicalsystem Biotechnology Co., Ltd (Ningbo, Zhejiang, China).

### Cell culture

Human aortic smooth muscle cells (HASMCs) were purchased from ATCC and cultured in complete DMEM medium. Human umbilical vein endothelial cells (HUVECs) and human aortic endothelial cells (HAECs) were purchased from ATCC and cultured in Vasculife basal medium (EC medium) containing VEGF Lifefactors kit. Cells less than 10 passages were used for experiments. All studies with HASMCs, HAECs, and HUVECs were approved by the Ethics Committee of Hefei University of Technology (HFUT20191008002) and adhered strictly to the Declaration of Helsinki Principle 2008.

### In vivo studies with mice

The protocols for *in vivo* study with mice were approved by the Ethics Committee of Hefei University of Technology (HFUT20191008001) and conform to the Guide for the Care and Use of Laboratory Animals published by NIH. The animal studies were reported in compliance with the ARRIVE guidelines. ApoE deficient (apoE^−/−^) mice were originally obtained from the Jackson Laboratory (Bar Harbor, Maine, USA) and bred in the Animal Center of Hefei University of Technology (Hefei, China).

To study the effect of U0126 on aortic neointima formation, carotid injury in apoE^−/−^ mice was performed as described [12]. Briefly, apoE^−/−^ mice (~8-week old, male) were divided into two groups (5 mice/group) and pre-fed high-fat diet (HFD, 21% fat and 0.5% cholesterol) or HFD containing U0126 (3 mg/kg) for two weeks. Mice were anesthetized by isoflurane inhalation and injected buprenorphine (0.1 mg/kg) subcutaneously for a preemptive analgesia. Carotid injury was then performed by right-carotid ligation with 6–0 silk suture. The left carotid artery was performed sham operation without ligation. After surgery, mice were continued the diet feeding for another 4 weeks. At the end of experiment, the mice were euthanized by i.p injection of an overdose of pentobarbital (500 mg/kg). Then, mouse left and right carotid arteries were collected and used to prepare 5-µm frozen sections. The sections were then used to detect neointima areas by hematoxylin and eosin (HE) staining. The images were captured and used to determine the neointima and media areas (µm^2^/section) using the Photoshop CS3 software. The serum was collected to determine triglyceride (TG), total cholesterol, LDL-cholesterol, and HDL-cholesterol levels using commercially available enzymatic kits purchased from Medicalsystem Biotechnology Co., Ltd (Ningbo, Zhejiang, China).

To study the effect of U0126 on atherosclerotic endothelium function, apoE^−/−^ mice were randomly divided into two groups (5 mice/group). The animals were fed HFD or HFD containing U0126 (3 mg/kg), respectively. After 16 weeks of treatment, mice were euthanized by i.p injection of an overdose of pentobarbital (500 mg/kg), followed by collection of mouse aorta which was used to prepare 5-µm frozen sections.

To determine the effect of U0126 on genes of contractile VSMCs, apoE^−/−^ mice (∼8 week old, male) were randomly divided into two groups (5 mice/group). The animals were fed HFD or HFD containing U0126 (3 mg/kg), respectively. All mice were performed carotid injury by right carotid ligation as described above. After 1 week, all the mice were anesthetized and euthanized for collection of right carotid artery to extract total RNA samples. Expression of SMA and SM22α was determined by quantitative real-time PCR (qRT-PCR) [10].

After collection of samples, all mice were sent back to the Animal Center of Hefei University of Technology, and incinerated by an environmental protection company.

### Determination of collagen and elastin content by Verhoeff-van Gieson (VVG) staining and Sirius red staining

The collagen and elastin content in carotid artery were determined by VVG and Sirius red staining [13]. After mounted with resinous mounting medium, photos were captured using a light microscope (Leica DM5000B).

### Immunofluorescent staining

Expression of RGS16, CXCL12, CXCR4, VCAM-1, SMA, MOMA2, CD31, and SM22α in aortic root or carotid artery was determined by immunofluorescent staining with the 5-µm frozen sections of the corresponding samples and primary antibodies as described [14].

Expression of RGS16 and CXCR4 in HUVECs was also determined by immunofluorescent staining. Briefly, cells were plated on coated glass slides in 20-mm dishes and cultured in the corresponding medium. At ~90% confluence, cells received the indicated treatment. After removal of the treatment medium from dishes, cells were fixed in 4% PFA for 20 min, washed twice with PBS for 10 min, and blocked with 2% BSA for 2 h at room temperature. Cells were then incubated with the corresponding primary antibody overnight at 4°C followed by incubation with FITC-conjugated goat anti-rabbit IgG for 50 min at 37°C. Cells were then stained with DAPI solution to detect nucleus. The slides were removed from dishes, and photographed with a fluorescence microscope (Leica). The mean fluorescent intensity (MFI) of immunofluorescent image was calculated as described [15].

### Transfection of miR-126-3p antagomir or ERK1/2 siRNA

HUVECs or HAECs in 6-well plates were transfected with control or miR-126-3p antagomir or control siRNA or ERK1/2 siRNA using Lipofectamine® RNAiMAX Transfection Reagent (Invitrogen). After 24 h of transfection, cells were switched into complete EC medium and cultured for another 24 h, then received treatment in serum-free medium for 18 h.

### Luciferase reporter assay

RGS16 3ʹUTR (from +740 to +2408) containing miRNA regulatory elements (MREs) of miR-126-3p was generated by RT-PCR with total RNA extracted from HUVEC, sense (5′-TGCACTCGAGGTCTCCACGGCAGTGAGGAAG-3′) and antisense (5′-ATTAGCGGCCGCTCATATTTGACTTTTGCT-3′) primers. The RGS16 3′UTR region with miR-126-3p MREs mutation was generated by PCR. After digestion, the polymerase-chain reaction product was subcloned into psiCHECK-2 vector with sequence confirmed. The luciferase reporters were named as pRGS16 or pRGS16-mut, respectively. HUVECs in 48-well plates were transfected with pRGS16 or pRGS16-mut. The cells were also cotransfected with negative control mimic or miR-126-3p mimic, or treated with U0126 (2 µM). After 24 h of transfection or treatment, cells were lyzed, and cellular lysate was used to determine Firefly and Renilla luciferase activity using the Dual-Luciferase Reporter Assay System (Promega, Madison, WI) [6].

### Western blot and qRT-PCR

After the indicated treatment, SMA, SM22α, RGS16, and CXCR4 protein expression in total cellular proteins were determined by Western blot [14]. Briefly, total protein was extracted from HASMC, HAEC, and HUVEC with the RIPA lysis buffer. The protein samples were then separated by SDS-PAGE and transferred onto NC membrane. The membranes were blocked with 5% skim milk in PBST for 1 h at room temperature. Then, according to the molecule weight of the target protein, the membrane was cut into smaller pieces prior to hybridization. The membrane was then separately incubated with specific primary antibody (anti-RGS16, CXCR4, SMA, SM22α, α-Tubulin, or GAPDH) overnight at 4°C. After washed with PBST for three times, the membrane was incubated with HRP-conjugated secondary antibodies for 1 h at room temperature. Membrane was either exposed to film followed by development with developing solution and fixing bath or captured with chemiluminescence imaging system (Qinxiang, ChemiScope 3300 Mini, China). The densitometric values of immunoreactive bands were measured using Image J. The density of target band was normalized to GAPDH or α-Tubulin in the corresponding sample to reduce variance. The Western blot was repeated for three times, and all uncropped images were presented in the Supplement Data.

After treatment, total RNA was extracted from tissue samples or cells using Trizol reagent (Invitrogen, Carlsbad, CA, USA). Levels of miR-126-3p in carotid artery tissue or cells were analyzed by quantitative miR stem-loop RT-PCR technology (Ambion) [6]. Levels of SMA and SM22α in carotid artery tissue or CXCL12 and CXCR4 in cells were analyzed by qRT-PCR. For qRT-PCR, miR-126-3p levels were normalized by U6 mRNA in the corresponding samples, and CXCL12, CXCR4, SMA and SM22α levels were normalized by GAPDH mRNA in the corresponding samples.

### Data analysis

All data were generated from at least three independent experiments (biological repeats) in which 3 technical repeats were performed for each biological repeat. The results were expressed as mean ± SD. The raw data were initially subject to a normal distribution analysis with SPSS software (1-sample K-S of non-parametric test). All the data in normal distribution were then analyzed by the unpaired Student’s T-test via GraphPad Prism software (version 7.0, GraphPad Software, San Diego, CA). The difference was considered significant if P < 0.05.

## Results

Endothelial dysfunction is an initial and essential step in vascular remodeling diseases, including atherosclerosis and neointima formation. We previously reported that MEK1/2 inhibitor, U0126, has a protective effect on the development of atherosclerosis and vascular calcification. However, the effect of MEK1/2 inhibitors on neointima formation and the underlying mechanism is not fully understood. The aim of current study was to disclose the effect of MEK1/2 inhibitor on neointima formation by activating miR-126-3p expression. Mechanistically, we investigated the potential role of MEK1/2 inhibitor in regulating miR-126-3p/RGS16/CXCL12/CXCR4 and miR-126-3p/VCAM1 axis.

### MEK1/2 inhibition reduces vascular remodeling

**Figure 1.**
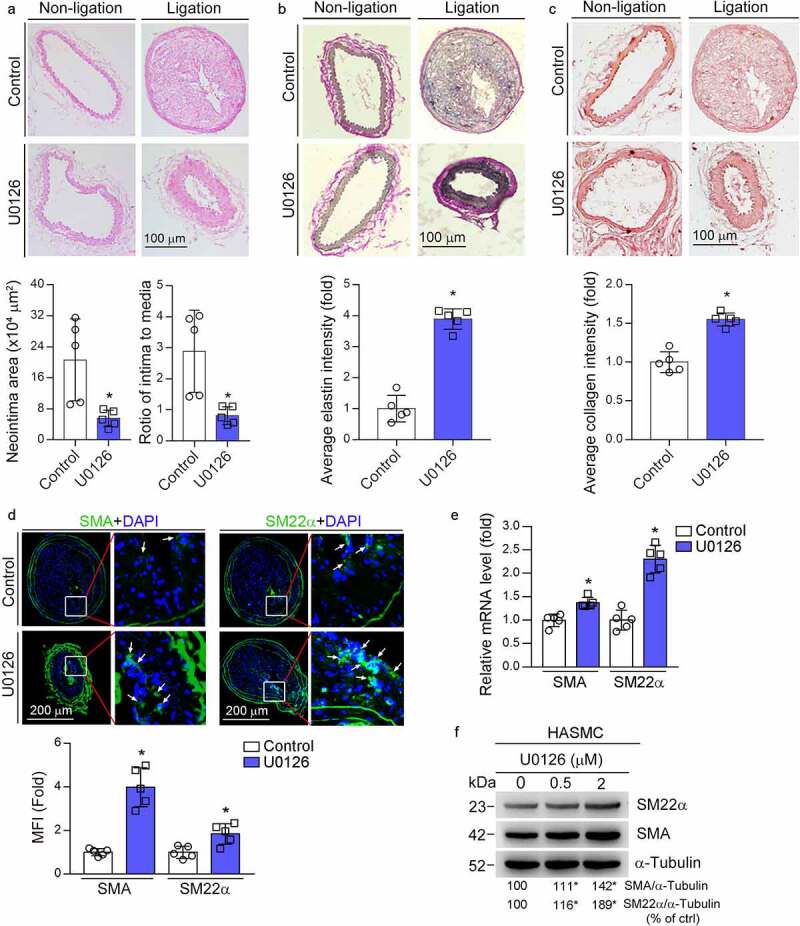
MEK1/2 inhibitor reduces carotid artery ligation induced neointimal formation. ApoE^−/−^ mice were pre-fed HFD or HFD containing U0126 (3 mg/kg) for 2 weeks. Mouse left and right carotid arteries were conducted sham and ligation operation respectively, followed by feeding of HFD or HFD plus U0126 for another 4 weeks. Both left and right carotid arteries were collected and prepared cross sections for the following assays: HE staining for morphological analysis with quantitative analysis of neointima and media areas (a); VVG staining for determination of elastin content (b), sirius red staining for determination of collagen content (c), the percentage of elastin or collagen positive area in whole intima was calculated, *P < 0.05 (n = 5). (d) expression of SMA and SM22α in right carotid artery was determined by immunofluorescent staining with quantitation of mean fluorescent intensity (MFI). While arrow: positive staining. *P < 0.05 (n = 5). (e) ApoE^−/−^ mice were conducted right carotid arteries ligation operation as described above. The mice were fed HFD or HFD plus U0126 for 1 week. Expression of SMA and SM22α mRNA in carotid artery was determined by qRT-PCR. *P < 0.05 (n = 5). (f) HASMCs were treated with U0126 at the indicated concentration for 16 h. Expression of SMA and SM22α protein was determined by Western blot. *P < 0.05 (n = 3).

It has been demonstrated the anti-atherogenic properties of MEK1/2 inhibitors [9,10,16]. However, the effect of MEK1/2 inhibitor on neointima formation and underlying mechanisms were not fully understood. U0126 is a specific MEK1/2 inhibitor broadly used in scientific researches. To define the detailed actions of U0126 on vascular remodeling, we determined the effect of U0126 on the partial ligation-induced neointima formation in carotid artery. In control mice, compared to left carotid artery with sham operation, a severe neointima was observed in right carotid artery after ligation. In contrast, U0126 substantially inhibited neointima formation (Figure 1(a)). The results of quantitative analysis show that U0126 substantially reduced neointima area (5.52 *vs*. 20.62) and ratio of intima to media area (0.81 *vs*. 2.89) (bottom panel, Figure 1(a)).

In control mice, the Verhoeff-van Gieson (VVG) staining shows little and disturbed collagen or elastin presented in the neointima area. In contrast, U0126 treatment clearly increased collagen/elastin content with a tight arrangement of elastic fibers lining within the neointima layer (Figure 1(b)). The results of Sirius red staining further confirmed that U0126 treatment increased collagen content in the neointima area (Figure 1(c)). These data indicate that MEK1/2 inhibitors can increase vascular integrity and maintain vascular function. Correspondingly, U0126 increased content of contractile SMCs in the neointima areas since expression of smooth muscle α-actin (SMA) and smooth muscle protein 22α (SM22α), the markers for contractile SMCs, were increased (Figure 1(d)). We further confirmed that U0126 increased SMA and SM22α mRNA expression in the carotid artery (Figure 1(e)). Moreover, U0126 also increased SMA and SM22α protein expression in HASMC (Figure 1(f)). Taken together, Figure 1 suggests that U0126 inhibited vascular remodeling.

### MEK1/2 inhibitor attenuates the expression of endothelial RGS16 to activate CXCL12/CXCR4 pathway by enhancing miR-126-3p expression

**Figure 2.**
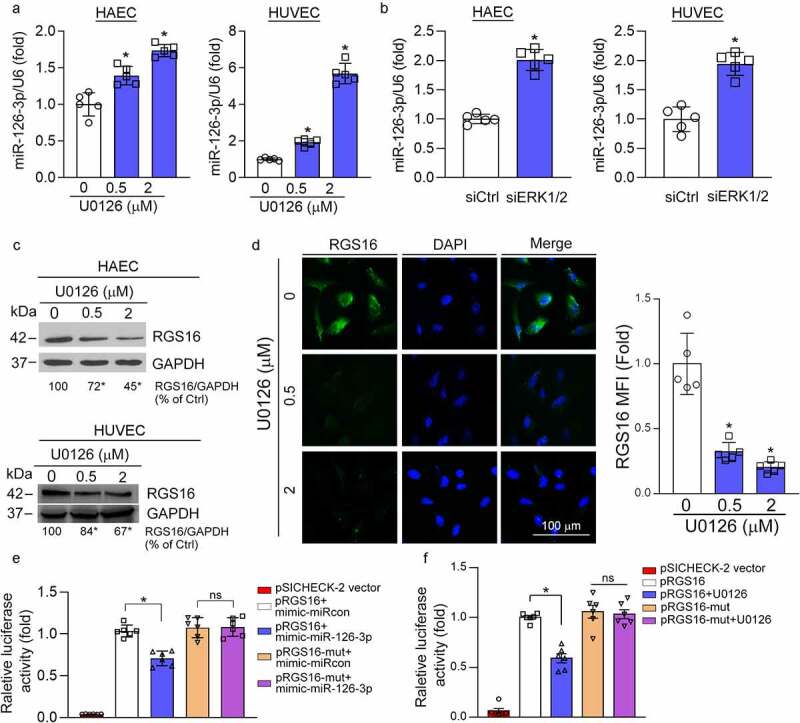
MEK1/2 inhibition attenuates expression of RGS16 by enhancing miR-126-3p expression in endothelial cells. (a, c, d) HAEC and HUVEC were treated with U0126 at the indicated concentration for 16 h. (a) Total RNA was extracted and used to determine miR-126-3p expression. *P < 0.05 (n = 5). (c) Total proteins were extracted, and the protein level of RGS16 was detected by Western blot. *P < 0.05 (n = 3). (d) expression of RGS16 in HUVEC was also detected by immunofluorescence assay. *p < 0.05 (n = 5). (b) HAEC or HUVEC in a six-well plate were transfected with control siRNA (siCtrl) or ERK1/2 siRNA (siERK1/2) for 24 h in serum-free medium, followed by switching the cells into complete medium to culture for 48 h. Total RNA was extracted and used to determine miR-126-3p expression. *P < 0.05 (n = 5). (e, f) The wild type RGS16 3′UTR or the 3ʹUTR with miR-126-3p MREs mutation was generated by PCR. After digestion, the polymerase chain reaction product was subcloned into psiCHECK-2 vector with sequence confirmed. The luciferase reporters were named as pRGS16 or pRGS16-mut, respectively. HUVECs in 48-well plates were transfected with pRGS16 or pRGS16-mut. The cells were also cotransfected with negative control mimic/miR-126-3p mimic (e) or treated with U0126 (f). After 24 hours of transfection or treatment, cells were lyzed, and cellular lysate was used to determine Firefly and Renilla luciferase activity. *P < 0.05, ns: not significant (n = 6).

The excessive proliferation of SMCs and ECs is the main cause of intima hyperplasia. However, we previously determined that U0126 had no effect on HASMC viability, cell cycle, and apoptosis [10]. Therefore, the U0126-reduced neointima formation may be related with ECs. Our previous study shows that ERK1/2 inhibition increases endothelial miR-126-3p expression [10]. RGS16 is a direct target gene of miR-126-3p. Related studies have shown that apoptotic endothelial cells can release apoptotic bodies containing miR-126-3p. These apoptotic bodies can act on neighboring endothelial cells, and promote expression of CXCR4/CXCL12 by inhibiting RGS16, which plays a role in enhancing endothelial repair [6]. Therefore, we initially verified that U0126 or ERK1/2 siRNA can up-regulate the expression of miR-126-3p in HAEC and HUVEC (Figure 2(a,b)). Accordingly, we detected the effect of U0126 on RGS16 expression in HAEC and HUVEC, and found that U0126 inhibited RGS16 expression in a concentration-dependent manner (Figure 2(c)). Moreover, we confirmed the inhibitory effect of U0126 on RGS16 expression by immunofluorescence staining in HUVEC (Figure 2(d)). It has been reported that RGS16 is a direct target gene of miR-126-3p^6^. To clarify it, we cloned the wild-type RGS16 3ʹUTR and the 3ʹUTR with miR-126-3p binding site mutation. We found that mimic-miR-126-3p reduced pRGS16 promoter activity. However, mutation of the miR-126-3p binding site in RGS16 3ʹUTR (pRGS16-mut) blocked the inhibitory effect of mimic-miR-126-3p on pRGS16 promoter activity (Figure 2(e)). Moreover, U0126-inhibited pRGS16 promoter activity was also abolished in pRGS16-mut transfected cells (Figure 2(f)).

**Figure 3.**
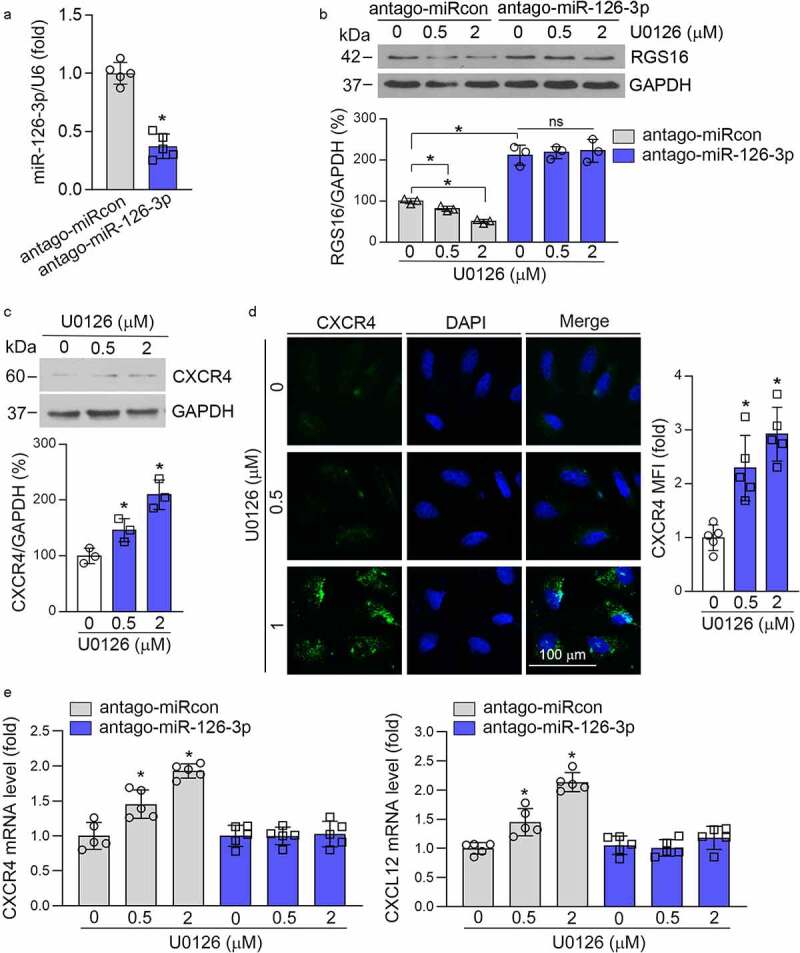
MEK1/2 inhibition reduces RGS16 to activate CXCR4/CXCL12 signaling pathways in endothelial cells via activating miR-126-3p. (a, b, e) HUVEC were transfected with control (miRCon) or miR-126-3p antagomir for 24 h. Cells were switched to complete EC medium and continued culture for another 24 h, then received U0126 treatment at the indicated concentrations overnight. Expression of miR-126-3p was determined by qRT-PCR (a). *P < 0.05 **(n = 5)**. Expression of RGS16 was detected by Western blot (b). *P < 0.05 (n = 3). Expression of CXCR4 and CXCL12 was detected by qRT-PCR (e) . *P < 0.05 (n = 5). (c, d) HUVEC was treated with U0126 at the indicated concentration for 16 h. Expression of CXCR4 was detected by Western blot (c) and immunofluorescence staining (d). *P < 0.05 (n = 3 in C, and n = 5 in D).

In addition, neutralizing miR-126-3p in HUVEC by miR-126-3p antagomir (Figure 3(a)) increased RGS16 expression (Figure 3(b)), and abolished U0126-inhibited RGS16 expression (Figure 3(b)). Thus, inhibition of RGS16 expression by U0126 is mediated by activated miR-126-3p. RGS16 can negatively regulate the expression of CXCR4, thereby affecting the function of CXCR4/CXCL12. Indeed, U0126 increased CXCR4 expression in HUVEC (Figure 3(c,d)). In addition, U0126-induced CXCL12 and CXCR4 expression was abolished by miR-126-3p antagomir (Figure 3(e)). The above results indicate that MEK1/2 inhibitor affect RGS16/CXCL12/CXCR4 signaling pathway via induction of miR-126-3p expression.

### MEK1/2 inhibitor attenuates the expression of RGS16 to activate CXCL12/CXCR4 pathway in vivo

**Figure 4.**
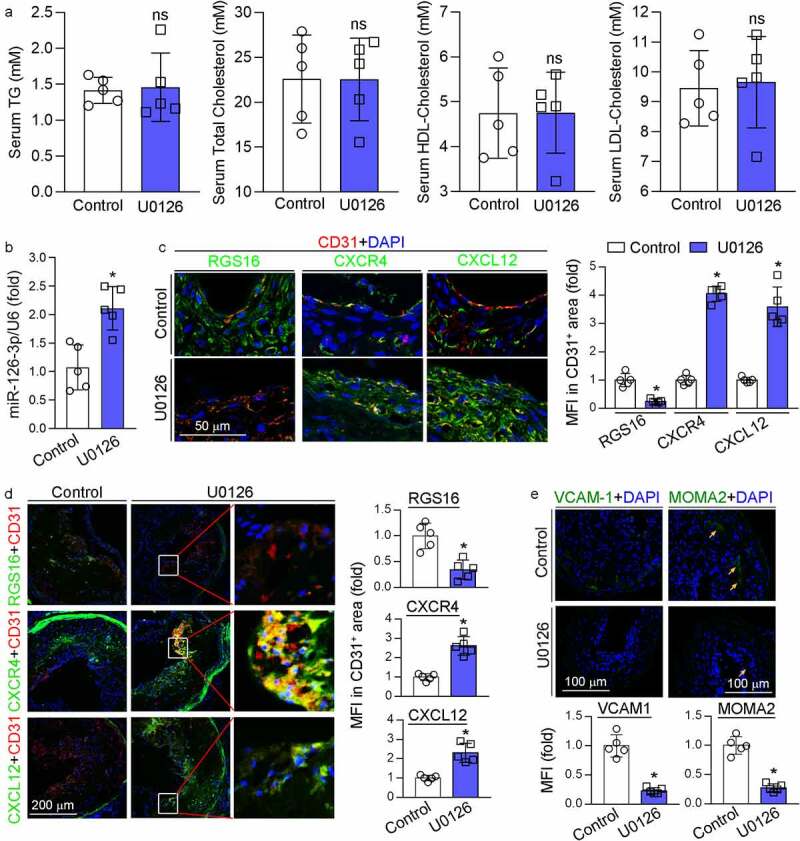
MEK1/2 inhibition facilitates endothelium repair *in vivo*. (a-c, e) carotid artery samples collected from mice in Figure 1 were used for the following assays: serum TG, total cholesterol, LDL-cholesterol and HDL-cholesterol levels (a) were determined by kits. *P < 0.05 (n = 5); miR-126-3p levels in carotid artery (b) were determined by qRT-PCR. *P < 0.05 (n = 5); expression of RGS16, CXCR4, CXCL12, CD31, VCAM-1 and MOMA2 was determined by immunofluorescent staining (c, e). RGS16, CXCR4, CXCL12 MFI in CD31-positive area were quantitative analyzed (right panel, **C**); MFI of VCAM-1 and MOMA2 were quantified respectively. *P < 0.05 (n = 5). (d) apoE^−/−^ mice (5/group) were fed high-fat diet (HFD) or HFD containing U0126 (3 mg/kg) for 16 weeks. Expression of RGS16, CXCR4, CXCL12, CD31 in aortic root were determined by immunofluorescent staining and quantitatively analyzed of double-positive area. *P < 0.05 (n = 5).

We determined the levels of triglyceride, total cholesterol, HDL cholesterol and LDL cholesterol in mouse serum and observed no significant differences between control and U0126-treated groups, suggesting that U0126 had little effect on serum lipid profiles (Figure 4(a)), indicating the U0126-inhibited neointima formation was unrelated with serum lipid levels. We further determined if the protective effect of U0126 on neointima formation was related with RGS16-CXCR4/CXCL12 pathway. In mice with ligation operation, U0126 increased miR-126-3p expression in the carotid artery (Figure 4(b)). U0126 substantially reduced RGS16 expression in neointima areas, and consequently activated CXCR4 and CXCL12 expression in the neointima areas including the area rich of CD31-positive cells (Figure 4(c)). Additionally, we previously determined that the long-term U0126 treatment increased circulating miR-126-3p and miR-126-3p expression in aortas in apoE^−/−^ mice, implying U0126 may counteract endothelial injury through miR-126-3p actions [10]. Indeed, U0126 also significantly reduced RGS16 while increased CXCR4 and CXCL12 expression in lesion areas in HFD-fed apoE^−/−^ mice including the area containing CD31-positive cells (Figure 4(d)). Furthermore, reduction of VCAM-1 expression in the ligated carotid artery by U0126 (left panel, Figure 4(e)) was associated with decreased MOMA2 positive cells (right panel, Figure 4(e)) in the neointima area. Therefore, accumulation of monocyte/macrophages in mouse arterial wall may be reduced by U0126.

Taken together, the data in Figure 3 and Figure 4 indicate that U0126 can inhibit endothelial injury by activating CXCL12/CXCR4 pathway through suppression of RGS16 and VCAM-1 expression, which is linked to activated miR-126-3p expression.

## Discussion

Vascular intimal hyperplasia, occurring during the development of vascular diseases, is mainly caused by vascular injury. Its pathological process mainly includes endothelial cell activation and proliferation, monocyte adhesion and infiltration, VSMC phenotype switching/migration and proliferation, which leads to vascular intimal hyperplasia and vascular lumen stenosis. In the current study, we determined that MEK1/2 inhibitor reduced neointima formation. We previously demonstrate that MEK1/2 inhibitor inhibits SMC phenotype switching to the proliferative or osteogenesis phenotype while maintains it to the contractile phenotype [9,10]. Consistently, we determined that MEK1/2 inhibitor increased SMA and SM22α expression in the neointimal area. It has been proved that activation of ERK1/2 and p38 MAPK signaling pathways leads to SMCs proliferation and migration [17]. We determined that MEK1/2 inhibitor has no effect on HASMC proliferation, cell cycle, and apoptosis, but arrests HUVEC cell cycle in G1 phase [10]. Therefore, the MEK1/2 inhibitor-reduced vascular injury and intimal hyperplasia may be related with both SMC phenotype switching and EC activation/proliferation.

Multiple miRNAs control the function of cardiovascular cells, and are closely associated with cardiovascular diseases [18]. For instance, miR-92a was upregulated by the combination of low shear stress and atherogenic ox-LDL. *In vivo*, miR-92a specific antagomir reduced endothelial inflammation and the development of atherosclerosis in LDLR^−/−^ mice [19]. MiR-663 markedly suppressed the neointimal lesion formation in mice after carotid artery ligation-induced vascular injury by targeting the transcription factor JunB to reduce its downstream molecules, such as myosin light chain 9 and matrix metalloproteinase 9^11^. In addition, miR-22-3p induces VSMC differentiation via inhibiting the expression of TET2, a master regulator of VSMC phenotypic switching [20,21]. Increased miR-96-5p would ameliorate acute myocardial infarction-associated cardiomyocytes injury by targeting BCL2L13, and miR-96-5p may function as a potential diagnostic biomarker for patients with coronary artery disease [22]. Studies have shown that miR-126-3p is necessary for blood vessel development. Knockout of miR-126-3p expression in zebrafish and mice leads to loss of vascular integrity during embryonic development, which leads to vascular leakage and bleeding, and this effect is mainly achieved by direct inhibition of Sprouty-related EVH1 domain-containing protein 1 (SPRED1) and phosphoinositol-3 kinase regulatory subunit 2 (PIK3R2) by miR-126-3p [23]. Clinically, it has been found that the level of miR-126-3p in the circulatory system of patients with coronary artery disease and type 2 diabetes mellitus is decreased [24,25]. Moreover, miR-126-3p inhibits the expression of VCAM-1 in endothelial cells, thereby reducing the adhesion of monocytes to endothelial cells [26], which was also confirmed by our previous study [10]. Herein, similar results were also observed. MEK1/2 inhibitor inhibited VCAM-1 expression and subsequence MOMA2-positive cell accumulation within neointima area via upregulating miR-126-3p expression.

Endothelium dysfunction is an initial and essential step in the development of vascular remodeling including restenosis and atherosclerosis [27]. In the circumstance of atherosclerosis, apoptosis of endothelial cells will lead to the release of apoptotic bodies, which are rich in miR-126-3p. Those miR-126-3p-rich apoptotic bodies can then promote the mobilization and incorporation of Sca-1+ progenitor cells to facilitate endothelium repair [6]. Mechanically, miR-126-3p can inhibit the expression of RGS16, the negative regulator of CXCR4, thus activating the CXCR4 signaling pathway, leading to up-regulation of CXCR4 and CXCL12 expressions. Consistent with our previous study, we show that MEK1/2 inhibitor could significantly up-regulate the expression of miR-126-3p in endothelial cells and carotid artery in mice. Mechanistically, we previously demonstrate that ERK1/2 inhibition increases AMPKα phosphorylation in HUVECs, which further induces p53 phosphorylation at serine 15. The phosphorylated p53 can interact with the Drosha-p68 complex to enhance miR-126 precursor maturation [10]. Consequently, the expression of RGS16 was inhibited while CXCR4 and CXCL12 were increased by MEK1/2 inhibitor both *in vitro* and *in vivo*. However, Shi et al. demonstrated that TGFβ-Smad3 can induce CXCR4 expression to enhance vascular injury-induced intimal hyperplasia. Indeed, SMC CXCR4 knockout eliminated intimal hyperplasia, which was related with CXCR4-activited ERK1/2 activity [11]. It has been published recently that CXCL12 induced miR-126-3p expression, which enhanced CXCL12-induced pro-angiogenic effects, and SPRED-1 involved in miR-126-3p/CXCL12 axis-induced angiogenesis [28]. Studies also suggests that CXCL12/CXCR4 facilitates angiogenesis through activating ERK1/2 signal pathway [4,29]. CXCL12/CXCR4 also involved in the progression and invasion of multiple cancers [30–32]. In the contrary, Wang et al. demonstrated that miR-126-3p reduced palmitate-induced ERK1/2 phosphorylation in HUVEC. However, miR-126-3p had no effect on ERK1/2 phosphorylation in HUVEC at the basal level [33]. Those data suggest that miR-126-3p regulates ERK1/2 phosphorylation depending on the specific stimulus with the cells. In our study, the activity of ERK1/2 was abolished by MEK1/2 inhibitor. Therefore, the upstream regulator miR-126-3p, CXCR4, or CXCL12 was no longer functional on ERK1/2 activity. Moreover, the function of CXCR4/CXCL12 signaling pathway may be inconsistent in EC and SMC.

The effect of MEK1/2 inhibitor on macrophage inflammation and VSMC phenotype switching during neointima formation may also contribute to MEK1/2 inhibitor reduced neointima formation. We previously determined that MEK1/2 inhibitor increased macrophages interleukin-5 and interferon γ expression [14,34,35], and reduced foam cell formation by activating ATP binding cassette transporter A1 and G1 expression [16,36]. U0126 significantly suppressed the effect of scrapie responsive gene 1 on LPS‑induced chemokine CCL22 production. CCL22 is generally known to be chemotactic for monocytes, dendritic cells, natural killer cells, and chronically activated T lymphocytes, suggesting that MEK1/2 inhibitor may block infiltration of these cells [37].

However, there are some limitations to our study. First, we determined that MEK1/2 inhibitor reduced neointima formation only in wild-type mice but not miR-126 knockout mice. Second, MEK1/2 inhibitor may also affect macrophage and VSMC to reduce neointima formation. In subsequent experiments, miR-126-3p antagomir-injected or miR-126 endothelium knockout mice may be used to determine the effect of MEK1/2 inhibitor on neointima formation.

## Conclusions

In conclusion, we demonstrate that MEK1/2 inhibitor can inhibit intimal hyperplasia. Mechanistically, MEK1/2 inhibitor induced miR-126-3p expression in endothelial cells. The upregulated miR-126-3p directly inhibited RGS16 to activate CXCL12/CXCR4 signaling pathway, thereby facilitating endothelium repair. Additionally, miR-126-3p inhibited VCAM1 expression to reduce monocyte adhesion/infiltration. Moreover, MEK1/2 inhibitor may also promote VSMC contractile phenotype differentiation. Our data suggest that MEK1/2 inhibitor may be a novel therapy for patients with intimal hyperplasia, including restenosis and atherosclerosis.

## Supplementary Material

Supplemental Material

## Data Availability

The datasets used and/or analyzed during the current study are available from the corresponding author upon reasonable request.
