# Supplementary material for: MEK1/2 inhibitor inhibits neointima formation by activating miR-126-3p/ C-X-C motif chemokine ligand 12 (CXCL12)/C-X-C motif chemokine receptor 4 (CXCR4) axis
Source: Bioengineered. 2022 Apr 29;13(4):11214–27. doi: 10.1080/21655979.2022.2063496 (PMC9208476; doi:10.1080/21655979.2022.2063496)
Supplement: Supplemental Material [file KBIE_A_2063496_SM4447.docx]

<https://www.jianguoyun.com/c/sd/14d258e/16b72296dc9c25d0>

<https://www.jianguoyun.com/c/sd/14d2591/f3ce3ccb206f992>

<https://www.jianguoyun.com/c/sd/14d2597/3b9efa1a62002f44>
